# Loot Boxes, Gambling, and Problem Gambling Among Young People: Results from a Cross-Sectional Online Survey

**DOI:** 10.1089/cyber.2020.0299

**Published:** 2021-04-09

**Authors:** Heather Wardle, David Zendle

**Affiliations:** ^1^ School of Social and Political Sciences, University of Glasgow, Glasgow, United Kingdom.; ^2^ Faculty of Public Health and Policy, London School of Hygiene and Tropical Medicine, London, United Kingdom.; ^3^ Department of Computer Science, University of York, York, United Kingdom.

**Keywords:** gambling, young people, loot boxes, gaming

## Abstract

With the introduction of gambling-like features within video games (e.g., loot boxes) new forms of hybrid-gambling products have emerged, yet little is known about their relationship to gambling and problem gambling among those most likely to engage: young people. This article examines the relationship between the purchase of loot boxes, gambling behavior, and problem gambling among young people ages 16–24. Cross-sectional data were analyzed from wave 1 of the Emerging Adults Gambling Survey, an online survey of 3,549 people, aged 16–24. Data were weighted to reflect the age, sex, and regional profile of Great Britain. Measured included past-year purchase of loot boxes, engagement in 17 different forms of gambling (weekly, yearly, and weekly spend); and problem gambling status. Other covariates include impulsivity and sociodemographic status. Young adults who purchase loot boxes are more likely to be gamblers and experience problem gambling than others. In unadjusted regression models, the odds of problem gambling were 11.4 (95% confidence interval [CI] 7.6 to 16.9; *p* < 0.001) times higher among those who purchased loot boxes with their own money. This relationship attenuated but remained significant (odds ratio 4.5, 95% CI 2.6–7.9) when gambling participation, impulsivity, and sociodemographic factors were taken into account. The purchase of loot boxes was highly associated with problem gambling, the strength of this association being of similar magnitude to gambling online on casino games or slots. Young adults purchasing loot boxes within video games should be considered a high-risk group for the experience of gambling problems.

## Introduction

Recent years have seen an emerging trend of gambling-like features being embedded in different contexts.^1^ This is especially so within video games and is, arguably, best exemplified by the growth of loot boxes within video games.^2,3^ Loot boxes are items that may be bought for real-world money, but which contain randomized contents whose value is uncertain at the point of purchase.^4^ They are a popular form of microtransaction now included within video games to obtain money from players, upon which game developers are increasingly reliant as a revenue stream. Indeed, recent research has suggested that the majority of top-grossing mobile games on both Apple and Android devices now contain loot boxes.^4^

Similarly, a recent analysis of the desktop gaming platform *Steam* investigated the proportion of desktop play sessions that take place in games with loot boxes. It has suggested that more than 70 percent of desktop play sessions now take place in a game that is monetized through loot boxes.^5^

Loot boxes, along with other microtransaction processes, have been described as a “predatory practice,” which entraps people into repeated purchasing.^2^ It has been suggested that they are “psychologically akin” to gambling as individuals stake money on the uncertain outcome of a future event in the hope of receiving something of greater value.^3^ Some jurisdictions agree and have taken regulatory action: Belgium has banned the use of loot boxes within some video games stating they are a violation of gambling legislation; gambling authorities in the Netherlands have ruled that some loot boxes constitute unlicensed games of chance; and China has required that the odds of winning be displayed to consumers.

There is some evidence that consumers themselves view loot box purchase as a form of gambling. In two separate small-scale surveys in Canada, between 68 percent and 86 percent of participants agreed that loot boxes were a form of gambling and between 75 percent and 79 percent of participants agreed that opening a loot box felt like making a bet.^6^ In Great Britain, a recent study by the Royal Society for Public Health found that 79 percent of young people ages 11–24 thought that loot boxes were a highly addictive form of gambling.^7^

An emerging evidence base has demonstrated an association between the purchase of loot boxes and problem gambling, with these findings repeated across time and space despite studies using different methodologies.^7–13^ However, the majority of these studies simply look at the association between loot box purchasing and problem gambling and do not take into the broader gambling or gaming behaviors of these people. Gainsbury^1^ has noted the need for caution, arguing that observed relationships between loot box engagement and problem gambling may be explained by a confound: interest in both gambling and gaming.

Similar arguments occur within gambling studies, whereby it is postulated that the relationship between specific types of gambling and problem gambling are confounded by wider interest and engagement in gambling itself: the “involvement” hypothesis.^14^ Some studies have supported this, finding that once the depth and breadth of gambling engagement are taken into account, the relationship between specific gambling activities and problem gambling attenuates or is no longer significant.^14,15^ Others have found that the relationship persists for certain activities.^16^

Gambling involvement is just one of many possible confounds which may explain an association between loot box purchase and problem gambling. Other potential confounds include personality traits, such as impulsivity or sociodemographic/economic status. Impulsivity has been identified as having a strong relationship with problem gambling in many other studies.^17^ One study has suggested that impulsivity was related to both loot box purchasing and problem gambling. However, no investigation of whether impulsivity accounted for the associations between the two took place. In addition, shared features of the sociodemographic or economic profile of loot box purchasers and problem gamblers may also explain associations between loot box engagement and gambling. Being male, younger, from non-white ethnic groups, and unemployed are factors commonly associated with problem gambling.^18^

The aims of this study were to investigate the relationship between loot box purchasing and problem gambling within a high-quality online panel survey of 16–24-year olds. Specific objectives were to:

(a) Explore the association between loot box purchase and problem gambling among those 16–24 years of age
(b) Examine the extent to which any evident association is accounted for by shared sociodemographic, economic, or personality traits such as impulsivity
(c) Explore if any observed relationship between loot box purchase is attenuated or explained by engagement in other gambling activities to test the gambling involvement hypothesis.

## Methods

### Data collection

The Emerging Adult's Gambling Survey collected data from 3,549 16–24-year olds. Participants were drawn from YouGov's online panel of over 1 million people living in Britain.^19,20^ This has up-to-date information on the profile of each member, allowing subsets of panel members to be invited to participate according to certain characteristics. For this study, participants were eligible if they were between 16 and 24 years of age, living in Britain, and had not taken part in any other YouGov study on gambling in the past year.

Email invites to participate were sent by YouGov to a random selection of their panel members, stratified by region. This email asked them to take part in a survey, without advertising its content, and asked participants to click through to the bespoke study. The first page of the bespoke survey then described the aims and objectives of the survey and obtained consent. Ninety-three percent of people who accessed this page went on to complete the survey. Data were collected between June and August 2019.

The survey asked about gambling, gaming, social media, and health-related behaviors. The questionnaire was developed by the lead author and reviewed by an expert panel of academics. A field pilot collected data from 62 participants in May 2019. Pilot responses were reviewed by the lead author and members of the YouGov team and changes agreed. The first 250 responses for the main data collection were further reviewed for consistency, accuracy of routing, and to establish timing thresholds for seriousness checks. Participants who completed the survey in more than one standard deviation of the mean completion time were removed: 39 participants were excluded from the final dataset for this reason. Missing data were minimal and excluded from analyses (except where explicitly stated).

Ethics approval for the study was granted by London School of Hygiene and Tropical Medicine's Ethics Review Panel (ref: 16023).

### Measures

#### Gambling measures

Participants were asked to report whether they have ever gambled, and if so how often they gambled, on a range of 17 different gambling activities legally available in Great Britain. Those who had gambled on each activity in the past year, the past month, and the past week were then identified. Weekly gamblers were asked how much they had spent on gambling in the past 7 days. Problem gambling was measured using the Problem Gambling Severity Index (PGSI), a validated tool for the identification of gambling problems.^21^ The PGSI produces a score ranging from 0 to 27; a score of 0 indicated nonproblem gambling or nongambling; 1–2 is low-risk gambling; 3–7 is moderate-risk gambling and a score of 8 or more is indicative of problem gambling. Within this sample, the PGSI had strong internal consistency (Cronbach's α = 0.94) and correlated as expected other similar outcome measures (e.g., wellbeing and suicidality).

#### Gaming measures

A suite of questions adapted from the UK Gambling Commission's Youth Gambling Survey asked whether participants played video games in the last year and if so whether they had used their own money to open loot boxes in the last year.^22^

#### Other measures

Impulsivity was measured using a shortened form of the Eysenck impulsivity scale, which is validated for use among adolescents.^23,24^ Participants were asked to respond on a five-point scale how true seven different impulsive statements are for them. Impulsivity scores are computed as the average of the seven questions (mean = 2.6 [standard deviation 0.87]), similar to other published reports among young people.^25^ Ethnicity was captured using the Office of National Statistics' standardized ethnicity question. Because of low base sizes, responses were grouped into White/White British; Asian/Asian British; Black/Black British; Other. Age was captured in single age years and grouped into those ages 16–18; 19–21, and 22–24. Participants were asked whether they were employed (full time, part time, self employed) or in education or training to identify those not in education, employment, or training.

#### Analysis

Bivariate analysis compared the sociodemographic profile and gambling behaviors of those who purchased loot boxes in the past year with those who did not.

Multivariable binary logistic regression analyses were conducted with past problem gambling status entered as the dependent variable and past year loot box purchase as the independent variable to examine their association. To investigate how the association was affected by the inclusion of different controls, different blocks of variables were added sequentially to a series of regression models. Model 0 (the crude model) contained loot box purchase alone; model 1 added age, sex, and ethnicity; model 2 added impulsivity; and model 3 added past-year participation in the 17 individual gambling activities. Because gambling involvement can be captured in different ways, five variants of model 3 were run, using different measures of gambling involvement (whether a weekly gambler; number of gambling activities undertaken in the past year; weekly spend on gambling; frequency of gambling).

With the exception of impulsivity scores, all variables included in the models were categorical. Missing data were minimal and therefore excluded, except for ethnicity, where data were missing for 159 cases and coded as a dummy category. Diagnostic checks on multicollinearity were conducted by calculating the variance inflation factors (VIF) of all independent variables, all had VIF values of less than 2.^26^

Bivariate analyses were conducted using SPSS v19 complex survey module and regression analysis were performed using the complex survey function in Stata v15 to adjust for weighted stratified survey design. These complex survey modules produce a Wald *F*-test as the default test of significance.^27^ For bivariate analyses, this assesses the extent to which the independent variable (prevalence of loot box purchase, for example) varies by the dependent variables (age or gender, for example) and is the test on which all *p*-values are based. Estimates were weighted to match the age, sex, and regional profile of Great Britain. Analyses used weighted data and controlled for complex survey design; true (unweighted) bases are presented.

## Results

Interviews were conducted with 3,549 young people aged 16–24, of whom 42.5 percent (95% confidence interval [CI]: 40.9–44.1) had gambled on any activity in the past year, 3.7 percent (95% CI: 3.1–4.3) experienced problem gambling and 12.1 percent (95% CI: 11.1–13.3) had purchased loot boxes in the past year. Only buying scratchcards (19.1 percent, 95% CI: 17.8–20.4)) and lottery tickets (17.5 percent, 95% CI: 16.2–18.8) were more popular forms of gambling than the purchase of loot boxes.

As shown in Table 1, those who purchased loot boxes were more likely to be younger and were disproportionately more likely to be male than those who had not. The mean impulsivity scores of loot box purchasers were significantly higher than those who had not purchased them: 2.6 versus 2.2 (*p* < 0.001).

**Table 1.** Profile of Loot Box Purchasers

	Whether purchased loot boxes in past year	
	Yes	No
Sociodemographic characteristics		
Sex^**^		
Male	77.3%	47.7%
Female	22.7%	52.3%
Age group^**^		
16–18	38.9%	32.8%
19–21	29.4%	31.0%
22–24	31.8%	36.3%
Ethnic group		
White/White British	86.2%	86.0%
Black/Black British	3.0%	4.0%
South Asian	6.4%	7.8%
Mixed/Other	3.1%	1.6%
Gambling behaviors		
Whether a past-year gambler^**^	62.8%	39.7%
Whether a past-week gambler^**^	26.6%	4.8%
Mean spend (£) on gambling in past week^*^	£19.2	£5.5
Problem Gambling Severity Index Score^**^		
0: nonproblem gambling	61.0%	85.4%
1–2: low-risk gambling	14.8%	9.7%
3–7: moderate-risk gambling	7.2%	3.1%
8: problem gambling	16.9%	1.8%
Mean impulsivity score^**^	2.6	2.2
Bases (unweighted)	427	3,059

^*^ Variable significant at *p* < 0.05.

^**^ Variable significant at *p* < 0.01.

Loot box purchasers were also more likely to have gambled on any form of gambling in the past year (62.8 percent [95% CI: 58.2–67.4] versus 39.7 percent [95% CI: 38.0–41.4]; *p* < 0.01), to have spent more money on gambling in the past week (mean weekly expenditure £19.20 versus £5.50; *p* < 0.01) and were more likely to experience problem gambling than those who had not (16.9 percent [95% CI: 13.3–20.5] versus 1.8 percent [95% CI: 1.3–2.3]; *p* < 0.01). In short, loot box purchasers were highly engaged in other forms of gambling (see Supplementary Table S1 for Phi correlation coefficients between past-year loot box purchase and past-year engagement in other activities).

In the unadjusted (crude) regression model, the odds of problem gambling were 11.4 (95% CI: 7.6–16.9) times higher among those who had purchased loot boxes in the past year. When age, sex, and ethnicity were controlled for, the odds increased to 12.0 (95% CI: 7.7–18.7). Adding impulsivity to the model attenuated the odds, reducing to 9.0 (95% CI: 5.7–14.3). In the final, fully adjusted model where gambling participation variables were added, the odds attenuated further but remained significant (4.4, 95% CI: 2.4–7.8). The fully adjusted model included all other 17 forms for gambling activity individually, with betting online, betting in person with a boomaker, playing online bingo, and playing poker at a pub or club all having elevated odds of problem gambling. To check the sensitivity of these results, these models were also run on the subsample of gamblers, which gave broadly similar results (Supplementary Tables S2 and S3). Different measures of gambling involvement displayed the similar results, with the odds of problem gambling attenuating among loot box purchasers but remaining significant (Tables 2 and 3).

**Table 2.** Adjusted Odds Ratios for Problem Gambling

Whether purchased loot boxes in the past year	Descriptive statistics	Model 1			Model 2			Model 3		
	*n*^a^ (%)	OR	95% CI lower	95% CI upper	OR	95% CI lower	95% CI upper	OR	95% CI lower	95% CI upper
		*p* < 0.001			*p* < 0.001			*p* < 0.001		
No	3,072 (86.2)	1			1			1		
Yes	412 (12.1)	12.0	7.7	18.7	9.0	5.7	14.3	4.4	2.4	7.8
Unsure	65 (1.8)	5.7	2.3	14.2	4.4	1.8	10.9	8.0	3.3	19.6
Sex		*p* = 0.462			*p* = 0.463			*p* = 0.372		
Male	1,627 (51.3)	1			1			1		
Female	1,922 (48.7)	0.9	0.6	1.3	0.8	0.5	1.3	0.8	0.5	1.3
Age group		*p* < 0.05			*p* < 0.05			*p* = 0.543		
16–18	1,103 (33.4	1			1			1		
19–21	1,212 (31.0)	2.0	1.2	3.3	2.0	1.2	3.4	1.4	0.7	2.6
22–24	1,234 (35.6)	1.7	1.0	2.9	1.6	0.9	2.8	1.1	0.6	2.1
Ethnic group		*p* < 0.001			*p* < 0.001			*p* < 0.001		
White/White British	2,919 (81.9)	1			1			1		
Asian	258 (7.2)	2.3	1.2	4.6	2.0	1.0	4.0	2.5	1.0	6.4
Black	58 (1.7)	4.7	2.2	10.0	4.7	2.0	11.1	5.7	1.8	17.9
Mixed/Other	162 (4.5)	5.2	2.7	9.9	4.6	2.2	9.8	5.6	2.5	12.3
Unknown	159 (4.6)	3.0	1.3	6.6	3.2	1.5	7.2	4.4	2.1	9.0
Economic status		*p* = 0.835			*p* = 0.596			*p* = 0.229		
In education, employment, or training	3,107 (87.4)	1			1			1		
Not in education, employment, or training	442 (12.6)	1.1	0.6	1.9	1.2	0.6	2.2	1.5	0.8	3.0
Impulsivity					*p* < 0.001			*p* < 0.001		
Impulsivity score	Mean score: 2.35				2.9	2.4	3.5	2.6	2.1	3.3
Past year participation in^b^:										
Lotteries	629 (17.5)							0.7	0.3	1.6
Scratchcards	662 (19.1)							1.6	0.7	3.7
Slot machines	217 (6.2)							1.7	0.8	3.5
Machines in bookmakers (formerly fixed odd betting terminals)	66 (2.1)							1.9	0.7	5.3
Betting on online^**^	521 (14.9)							2.5	1.3	4.8
Gambling on online casino games or slots	138 (4.0)							2.2	0.9	5.6
Gambling on online bingo	68 (1.8)							1.7	0.4	7.6
Betting at a bookmakers^**^	258 (7.1)							4.8	2.4	9.6
Playing casino games at a casino	101 (2.9)							0.2	0.0	1.0
Playing bingo at a club^*^	205 (5.4)							2.2	1.0	4.9
Football pools	93 (2.7)							1.2	0.4	3.3
Playing poker at a pub/club^**^	46 (1.3)							10.8	3.1	36.9
Private betting or gambling with friends, family or colleagues	333 (9.7)							0.5	0.1	1.5

^a^ Bases are unweighted while proportions are weighted.

^b^ All odds are presented relative to the reference category of having not participated in each activity in the past year.

^*^ *p* < 0.05.

^**^ *p* < 0.01.

CI, confidence interval; OR, odds ratio.

**Table 3.** Adjusted Odds Ratios for Problem Gambling—with Different Controls for Gambling Involvement

	Descriptive statistics	Model 1			Model 2			Model 3a			Model 3b			Model 3c			Model 3d		
	*n*^a^ (%)	OR	95% CI lower	95% CI upper	OR	95% CI lower	95% CI upper	OR	95% CI lower	95% CI upper	OR	95% CI lower	95% CI upper	OR	95% CI lower	95% CI upper	OR	95% CI lower	95% CI upper
Whether purchased loot boxes in the past year		*p* < 0.001			*p* < 0.001			*p* < 0.001			*p* < 0.001			*p* < 0.001			*p* < 0.001		
No	3,072 (86.2)	1			1			1			1			1			1		
Yes	412 (12.1)	12.0	7.7	18.7	9.0	5.7	14.3	4.0	2.3	6.8	8.8	5.5	14.1	5.5	3.3	9.5	3.2	1.9	5.4
Unsure	65 (1.8)	5.7	2.3	14.2	4.4	1.8	10.9	4.6	1.6	13.1	4.7	1.9	11.5	5.2	2.1	12.9	10.0	2.3	44.2
Sex		*p* = 0.462			*p* = 0.463			*p* = 0.568			*p* = 0.973			*p* = 0.563			*p* = 0.413		
Male	1,627 (51.3)	1			1			1			1			1			1		
Female	1,922 (48.7)	0.9	0.6	1.3	0.8	0.5	1.3	1.2	0.7	1.9	0.9	0.6	1.4	1.0	0.6	1.6	1.2	0.7	2.1
Age group		*p* < 0.05			*p* < 0.05						*p* = 0.256			*p* = 0.363			*p* = 0.265		
16–18	1,103 (33.4)	1			1			1			1			1			1		
19–21	1,212 (31.0)	2.0	1.2	3.3	2.0	1.2	3.4	1.4	0.8	2.4	2.1	1.2	3.5	1.4	0.8	2.5	1.2	0.7	2.2
22–24	1,234 (35.6)	1.7	1.0	2.9	1.6	0.9	2.8	0.9	0.5	1.6	1.7	1.0	3.0	1.1	0.6	2.0	0.8	0.4	1.4
Ethnic group		*p* < 0.001			*p* < 0.001			*p* < 0.001			*p* < 0.001			*p* < 0.001			*p* < 0.001		
White/White British	2,919 (81.9)	1			1			1			1			1			1		
Asian	258 (7.2)	2.3	1.2	4.6	2.0	1.0	4.0	2.2	0.9	5.1	2.0	1.0	4.1	2.6	1.2	5.5	2.6	1.1	6.1
Black	58 (1.7)	4.7	2.2	10.0	4.7	2.0	11.1	3.1	1.1	8.3	4.7	2.0	11.2	5.8	2.1	15.9	3.7	1.2	11.3
Mixed/Other	162 (4.5)	5.2	2.7	9.9	4.6	2.2	9.8	5.0	2.1	11.9	4.4	2.0	9.7	5.5	2.7	11.5	6.5	2.5	16.8
Unknown	159 (4.6)	3.0	1.3	6.6	3.2	1.5	7.2	3.8	1.7	8.6	3.2	1.4	7.3	4.7	2.1	10.7	4.6	1.8	11.5
Economic status		*p* = 0.835			*p* = 0.596						*p* = 0.445			*p* = 0.208			*p* = 0.192		
In education, employment, or training	3,107 (87.4)	1			1			1			1			1			1		
Not in education, employment, or training	442 (12.6)	1.1	0.6	1.9	1.2	0.6	2.2	1.3	0.7	2.5	1.0	0.5	2.0	1.5	0.8	2.9	1.6	0.8	3.1
Impulsivity					*p* < 0.001			*p* < 0.001			*p* < 0.001			*p* < 0.001			*p* < 0.001		
Impulsivity score	Mean score: 2.35				2.9	2.4	3.5	2.6	2.1	3.3	2.9	2.3	3.5	2.7	2.1	3.3	2.5	2.0	3.2
Past-week gambler								*p* < 0.001											
No	1,212 (80.5)							1											
Yes	284 (19.5)							19.9	11.9	33.3									
Total money spent gambling in last week											*p* = 0.290								
£ (nonweekly gamblers = £0)	Mean £ = £7.20										1.00	1.00	1.01						
Number of gambling activities in the past year														*p* < 0.001					
Number of gambling activities	Mean: 1.05													1.4	1.3	1.6			
Frequency of gambling																	*p* < 0.001		
Did not gamble in the past year/gambled once or twice a year	2,986 (83.6)																1		
Gambled monthly	208 (6.1)																18.2	6.8	48.6
Gambled fortnightly	100 (2.9)																35.9	12.2	105.6
Gambled weekly	138 (3.9)																48.7	19.2	123.4
Gambled more than weekly	117 (3.5)																79.4	29.1	216.4

^a^ Bases are unweighted while proportions/means are weighted.

## Discussion

Researchers have suggested that a relationship between problem gambling and loot box purchasing may be explained by confounding through third variables, such as gambling engagement, personality traits, or socioeconomic features.^1^ Our study aimed to examine this. While there was some attenuation in the association, these data did not support this point of view. Bivariate analysis showed that loot box purchasers were heavily engaged in other forms of gambling, yet the relationship between loot box purchasing and problem gambling remained substantial and significant even when this broader gambling involvement was statistically taken into account. In the fully adjusted model, the odds of problem gambling were 4.4 times higher among those who purchased loot boxes than those who had not.

The fully adjusted model included impulsivity as well as sociodemographic and economic characteristics. When entered separately in models 1 and 2, respectively, these factors showed limited attenuation of the association between loot box purchase and problem gambling. The greatest attenuation was observed when gambling involvement measures were entered into the models. Given this, it is clear that gambling consumption accounts for some of this relationship, but not all. Indeed, one may credibly argue that the odds ratio of 4.4 associated with our fully adjusted model underestimates the strength of links between problem gambling and loot box spending.

Gambling engagement is likely to covary with problem gambling. Research suggests the possibility for two potential causal processes to operate in this domain: an attraction effect (in which problem gamblers are more likely to purchase loot boxes) and cultivation effects (in which loot box purchasing drives problem gambling). Regardless of which of these models hold, problem gambling will necessarily covary with gambling engagement, and loot box spending will necessarily covary with both of these variables. Thus, by attributing a set amount of covariance within our model to gambling engagement, and factoring it out of our effect size calculations, we may essentially be controlling for our own outcome. It is credible that the “true” size of any relationship between loot box spending and problem gambling lies somewhere between the estimate obtained in our fully controlled model, and in our prior models.

In this study, a range of sensible potential confounds were incorporated into the models. As with any piece of cross-sectional research, it may also be the case that other unmeasured confounds are responsible for the observed relationships. However, it may also be that the purchase of loot boxes is an activity of itself that is strongly associated with problem gambling, despite increased interest in other forms of gambling among those who engage. Previous research has highlighted other behaviors, which share this feature.^14^ For example, in Great Britain, this was the case with engagement with Fixed Odd Betting Terminals. The strength and persistence of this association was instrumental in government deeming these products more harmful than others and taking regulatory action.

Finally, loot box purchasing among those 16–24 years of age displayed as strong an association with problem gambling as some other gambling activities. Notably, only five individual gambling (or gambling-like) activities were associated with problem gambling in the fully adjusted model; of which loot box purchase was one. In this respect, the purchase of loot boxes had a stronger relationship with problem gambling than many other forms of gambling – including playing slot machines or online betting. The strength of the association was similar to that observed for gambling on online casino or slot style games, activities which are increasing in prevalence among young people and have been highlighted as a cause for concern among academics and policy makers.

These results have implications for clinicians and policy makers alike. Clinicians should recognize the high degree of overlap between gaming and gambling behaviors, especially among those who use microtransactions like loot boxes within video games. This evidence suggests that this group of young people may be vulnerable to the experience of gambling problems. It is possible that gaming and gambling disorders may be comorbid for some.

In Britain, as elsewhere, there are no regulatory restrictions on loot boxes: no age restrictions, no codes of conduct, and they are not subject to any kind of product-based regulation over stakes or prizes or nature of the offering.^28^ In many jurisdictions, regulation of products varies based on their demonstrated level of harmfulness – typically measured by an activity's association with problem gambling.^29,30^ This study demonstrates, for the first time that the purchase of loot boxes are associated with elevated rates of problem gambling among young people even after higher levels of gambling consumption are taken into account. Data need to be triangulated with other studies and replicated among other age groups, among people recruited using different sampling designs, and in different jurisdictions. However, our results suggest that the purchase of loot boxes among those 16–24 years of age may rank as a more “harmful” form of gambling that needs appropriate regulatory attention.

This study has a number of limitations. The sample frame is an online panel survey with attendant issues of generalizability. However, when researching young adults, it has advantages in terms of sample coverage over probability methods, which systematically exclude certain segments of young people (household studies exclude students; institutional studies exclude those living in other circumstances). This study arguably also represents an advance on its predecessors, which as Gainsbury notes,^1^ have tended used self-selected samples from online platforms, such as Reddit or Amazon's Mechanical Turk. Both of these platforms present specific limitations when it comes to sample coverage. Reddit provides online bulletin boards for topical discussion by a highly engaged subset of the general population. Samples recruited from Reddit are consequently unlikely to represent the general population.^31^ Similarly, crowdsourcing platforms, such as Amazon Mechanical Turk, tend to provide samples featuring high levels of gambling engagement among respondents.^32^ The generalizability of results extrapolated from these samples is often not clear.

The study did not include questions about other types of microtransactions used in video games and so there is limited information with which to contextualize the patterns of gaming play among loot box purchasers. However, the study did distinguish between loot box purchases with real currency and those opened using points or in-game currency, which had been won rather than bought. The results from this study therefore represents a certain subset of gamer – those willing to use their own money to gamble on these products. As with any survey, data for all measures are self-reported and may be subject to recall bias and while the self-reported data collected by the PGSI gives a reasonable approximation of gambling problems but is, of course, not a clinical assessment. Finally, these data are cross-sectional and so cannot attribute causation.

This study demonstrates a substantial association between the purchase of loot boxes and problem gambling, which is not fully accounted for by other patterns of gambling consumption. If loot boxes are considered a form of gambling, according to our study, their association with problem gambling ranks as highly as gambling online on casino games or slots. The mechanisms underpinning this need to be better understood. However, at the very least, those purchasing loot boxes within video games should be viewed as a high-risk group for the experience of gambling problems.

## Supplementary Material

Supplemental data

Supplemental data

Supplemental data
