# Supplementary material for: Loot Boxes, Gambling, and Problem Gambling Among Young People: Results from a Cross-Sectional Online Survey
Source: Cyberpsychol Behav Soc Netw. 2021 Apr 9;24(4):267–74. doi: 10.1089/cyber.2020.0299 (PMC8064953; doi:10.1089/cyber.2020.0299)
Supplement: Supplemental data [file Supp_TableS1.docx]

| **Supplementary Table 1. Phi Correlation Coefficients between past year purchase of lootboxes and engagement in individual gambling activities** | |
| --- | --- |
|  | **Phi correlation coefficient** |
| **Past year gambling activity** |  |
| Lotteries | 0.062 |
| Scratchcards | 0.095 |
| Slot machines | 0.148 |
| Machines in bookmakers (formerly fixed odd betting terminals) | 0.225 |
| Betting on online | 0.193 |
| Gambling on online casino games or slots | 0.183 |
| Gambling on online bingo | 0.090 |
| Betting at a bookmakers | 0.138 |
| Playing casino games at a casino | 0.082 |
| Playing bingo at a club | 0.056 |
| Football pools | 0.146 |
| Playing poker at a pub/club | 0.129 |
| Private betting or gambling with friends, family or colleagues | 0.102 |
