# Supplementary material for: Loot Boxes, Gambling, and Problem Gambling Among Young People: Results from a Cross-Sectional Online Survey
Source: Cyberpsychol Behav Soc Netw. 2021 Apr 9;24(4):267–74. doi: 10.1089/cyber.2020.0299 (PMC8064953; doi:10.1089/cyber.2020.0299)
Supplement: Supplemental data [file Supp_TableS2.docx]

| **Supplementary Table 2. Adjusted odds ratios for problem gambling among past year gamblers only** | | | | | | | | | | |
| --- | --- | --- | --- | --- | --- | --- | --- | --- | --- | --- |
|  | **Descriptive statistics** | **Model 1** | | | **Model 2** | | | **Model 3** | | |
|  | N^a^ (%) | OR | 95% CI lower | 95% CI upper | OR | 95% CI lower | 95% CI upper | OR | 95% CI lower | 95% CI upper |
| **Whether purchased loot boxes in the past year** |  | p<0.001 | | | p<0.001 | | | p<0.001 | | |
| No | 1206 (80.4%) | 1 |  |  | 1 |  |  | 1 |  |  |
| Yes | 264 (17.7%) | 8.2 | 5.2 | 12.9 | 5.9 | 3.6 | 9.6 | 3.8 | 2.2 | 6.7 |
| Unsure | 26 (1.8%) | 6.1 | 2.0 | 18.3 | 3.9 | 1.2 | 12.2 | 6.8 | 2.1 | 21.9 |
| **Sex** |  | p=0.852 | | | p=0.745 | | | p=0.288 | | |
| Male | 730 (54.9%) |  |  |  | 1 |  |  | 1 |  |  |
| Female | 766 (415%) | 1.0 | 0.6 | 1.5 | 0.9 | 0.6 | 1.5 | 0.7 | 0.4 | 1.3 |
| **Age group** |  | p=0.338 | | | p=0.544 | | | p=0.838 | | |
| 16-18 | 397 (27.7%) | 1 |  |  | 1 |  |  | 1 |  |  |
| 19-21 | 506 (31.4%) | 1.4 | 0.8 | 2.3 | 1.2 | 0.7 | 2.1 | 1.0 | 0.6 | 1.9 |
| 22-24 | 593 (40.9%) | 1.0 | 0.6 | 1.7 | 0.9 | 0.5 | 1.6 | 0.9 | 0.5 | 1.6 |
| **Ethnic group** |  | p<0.001 | | | p<0.001 | | | p<0.001 | | |
| White/White British | 1291 (85.9%) | 1 |  |  | 1 |  |  | 1 |  |  |
| Asian | 62 (4.6%) | 4.1 | 2.0 | 8.4 | 4.4 | 2.0 | 9.4 | 4.0 | 1.2 | 12.7 |
| Black | 27 (1.9%) | 4.7 | 2.1 | 10.6 | 5.8 | 2.1 | 16.1 | 6.5 | 1.8 | 23.7 |
| Mixed/Other | 64 (4.0%) | 6.6 | 3.2 | 13.9 | 8.0 | 3.3 | 19.4 | 8.4 | 3.4 | 21.0 |
| Unknown | 52 (3.7%) | 4.2 | 1.6 | 10.7 | 5.6 | 2.3 | 14.0 | 5.0 | 2.1 | 11.9 |
| **Economic status** |  | p=0.438 | | | p=0.132 | | | p=0.132 | | |
| In education, employment or training | 1305 (90.1%) | 1 |  |  | 1 |  |  | 1 |  |  |
| Not in education, employment or training | 146 (9.9%) | 1.3 | 0.7 | 2.5 | 1.6 | 0.9 | 3.0 | 1.7 | 0.9 | 3.4 |
| **Impulsivity** |  |  |  |  |  | | |  | | |
| Impulsivity score | Mean score: 2.33 |  |  |  | 3.0 | 2.4 | 3.8 | 2.7 | 2.1 | 3.5 |
| **Past year participation in^b^:** |  |  |  |  |  |  |  |  |  |  |
| Lotteries* | 629 (41.2%) |  |  |  |  |  |  | 0.5 | 0.3 | 0.9 |
| Scratchcards | 662 (44.8%) |  |  |  |  |  |  | 0.8 | 0.5 | 1.4 |
| Slot machines | 217 (14.5%) |  |  |  |  |  |  | 1.3 | 0.7 | 2.4 |
| Machines in bookmakers (formerly fixed odd betting terminals) | 66 (4.9%) |  |  |  |  |  |  | 1.7 | 0.7 | 4.1 |
| Betting on online | 521 (35.0%) |  |  |  |  |  |  | 1.2 | 0.7 | 2.1 |
| Gambling on online casino games or slots* | 138 (9.5%) |  |  |  |  |  |  | 2.3 | 1.1 | 4.9 |
| Gambling on online bingo | 68 (4.3%) |  |  |  |  |  |  | 1.9 | 0.6 | 5.8 |
| Betting at a bookmakers** | 258 (16.7%) |  |  |  |  |  |  | 3.0 | 1.7 | 5.3 |
| Playing casino games at a casino | 101 (6.7%) |  |  |  |  |  |  | 0.4 | 0.1 | 1.2 |
| Playing bingo at a club* | 205 (12.6%) |  |  |  |  |  |  | 1.5 | 0.8 | 2.9 |
| Football pools | 93 (6.4%) |  |  |  |  |  |  | 1.1 | 0.5 | 2.7 |
| Playing poker at a pub/club** | 46 (3.0%) |  |  |  |  |  |  | 8.0 | 2.7 | 23.4 |
| Private betting or gambling with friends, family or colleagues* | 333 (22.9%) |  |  |  |  |  |  | 0.3 | 0.1 | 0.8 |

^a^ Bases are unweighted whilst proportions are weighted.

^b^ all odds are presented relative to the reference category of having not participated in each activity in the past year.

*p<0.05; **p<0.01
