# Supplementary material for: Loot Boxes, Gambling, and Problem Gambling Among Young People: Results from a Cross-Sectional Online Survey
Source: Cyberpsychol Behav Soc Netw. 2021 Apr 9;24(4):267–74. doi: 10.1089/cyber.2020.0299 (PMC8064953; doi:10.1089/cyber.2020.0299)
Supplement: Supplemental data [file Supp_TableS3.docx]

| **Supplementary Table 3. Model statistics for main logistic regressions reported: models 1-3, Table 2** | | | | | | | | | | | | | | | | | | | |
| --- | --- | --- | --- | --- | --- | --- | --- | --- | --- | --- | --- | --- | --- | --- | --- | --- | --- | --- | --- |
|  | **Model 1** | | | | | | **Model 2** | | | | | | | **Model 3** | | | | | |
|  | OR | SE | t | p-value | 95% CI lower | 95% CI upper | | OR | SE | t | p-value | 95% CI lower | 95% CI upper | OR | SE | t | p-value | 95% CI lower | 95% CI upper |
| **Whether purchased loot boxes in the past year** |  | | | | | | |  | | | | | |  | | | | | |
| No | 1 |  |  |  |  |  | | 1 |  |  |  |  |  |  |  |  |  |  |  |
| Yes | 12.0 | 2.7 | 11.1 | 0.0 | 7.7 | 18.7 | | 9.0 | 2.1 | 9.4 | 0.0 | 5.7 | 14.3 | 4.4 | 1.3 | 5.0 | 0.0 | 2.4 | 7.8 |
| Unsure | 5.7 | 2.7 | 3.8 | 0.0 | 2.3 | 14.2 | | 4.4 | 2.0 | 3.3 | 0.0 | 1.8 | 10.9 | 8.0 | 3.7 | 4.6 | 0.0 | 3.3 | 19.6 |
| **Sex** |  | | | | | | |  | | | | | |  | | | | | |
| Male | 1 |  |  |  |  |  | | 1 |  |  |  |  |  |  |  |  |  |  |  |
| Female | 0.9 | 0.2 | -0.7 | 0.5 | 0.6 | 1.3 | | 0.8 | 0.2 | -0.7 | 0.5 | 0.5 | 1.3 | 0.8 | 0.2 | -0.9 | 0.4 | 0.5 | 1.3 |
| **Age group** |  | | | | | | |  | | | | | |  | | | | | |
| 16-18 | 1 |  |  |  |  |  | | 1 |  |  |  |  |  |  |  |  |  |  |  |
| 19-21 | 2.0 | 0.5 | 2.7 | 0.0 | 1.2 | 3.3 | | 2.0 | 0.5 | 2.7 | 0.0 | 1.2 | 3.4 | 1.4 | 0.4 | 1.0 | 0.3 | 0.7 | 2.6 |
| 22-24 | 1.7 | 0.5 | 2.0 | 0.0 | 1.0 | 2.9 | | 1.6 | 0.5 | 1.7 | 0.1 | 0.9 | 2.8 | 1.1 | 0.4 | 0.3 | 0.8 | 0.6 | 2.1 |
| **Ethnic group** |  | | | | | | |  | | | | | |  | | | | | |
| White/White British | 1 |  |  |  |  |  | | 1 |  |  |  |  |  |  |  |  |  |  |  |
| Asian | 2.3 | 0.8 | 2.5 | 0.0 | 1.2 | 4.6 | | 2.0 | 0.7 | 1.9 | 0.1 | 1.0 | 4.0 | 2.5 | 1.2 | 1.9 | 0.1 | 1.0 | 6.4 |
| Black | 4.7 | 1.8 | 4.1 | 0.0 | 2.2 | 10.0 | | 4.7 | 2.1 | 3.6 | 0.0 | 2.0 | 11.1 | 5.7 | 3.3 | 3.0 | 0.0 | 1.8 | 17.9 |
| Mixed/Other | 5.2 | 1.7 | 5.1 | 0.0 | 2.7 | 9.9 | | 4.6 | 1.8 | 3.9 | 0.0 | 2.2 | 9.8 | 5.6 | 2.2 | 4.3 | 0.0 | 2.5 | 12.3 |
| Unknown | 3.0 | 1.2 | 2.7 | 0.0 | 1.3 | 6.6 | | 3.2 | 1.3 | 2.9 | 0.0 | 1.5 | 7.2 | 4.4 | 1.6 | 4.0 | 0.0 | 2.1 | 9.0 |
| **Economic status** |  | | | | | | |  | | | | | |  | | | | | |
| In education, employment or training | 1 |  |  |  |  |  | | 1 |  |  |  |  |  | 1 |  |  |  |  |  |
| Not in education, employment or training | 1.1 | 0.3 | 0.2 | 0.8 | 0.6 | 1.9 | | 1.2 | 0.4 | 0.5 | 0.6 | 0.6 | 2.2 | 1.5 | 0.5 | 1.2 | 0.2 | 0.8 | 3.0 |
| **Impulsivity** |  | | | | | | |  | | | | | |  | | | | | |
| Impulsivity score |  |  |  |  |  |  | | 2.9 | 0.3 | 10.5 | 0.0 | 2.4 | 3.5 | 2.6 | 0.3 | 8.1 | 0.0 | 2.1 | 3.3 |
| **Past year participation in^b^:** |  |  |  |  |  |  | |  |  |  |  |  |  |  |  |  |  |  |  |
| Lotteries* |  |  |  |  |  |  | |  |  |  |  |  |  | 0.7 | 0.3 | -0.8 | 0.4 | 0.3 | 1.6 |
| Scratchcards |  |  |  |  |  |  | |  |  |  |  |  |  | 1.6 | 0.7 | 1.1 | 0.3 | 0.7 | 3.7 |
| Slot machines |  |  |  |  |  |  | |  |  |  |  |  |  | 1.7 | 0.6 | 1.5 | 0.1 | 0.8 | 3.5 |
| Machines in bookmakers (formerly fixed odd betting terminals) |  |  |  |  |  |  | |  |  |  |  |  |  | 1.9 | 1.0 | 1.2 | 0.2 | 0.7 | 5.3 |
| Betting on online |  |  |  |  |  |  | |  |  |  |  |  |  | 2.5 | 0.8 | 2.6 | 0.0 | 1.3 | 4.8 |
| Gambling on online casino games or slots* |  |  |  |  |  |  | |  |  |  |  |  |  | 2.2 | 1.1 | 1.6 | 0.1 | 0.9 | 5.6 |
| Gambling on online bingo |  |  |  |  |  |  | |  |  |  |  |  |  | 1.7 | 1.3 | 0.7 | 0.5 | 0.4 | 7.6 |
| Betting at a bookmakers** |  |  |  |  |  |  | |  |  |  |  |  |  | 4.8 | 1.7 | 4.4 | 0.0 | 2.4 | 9.6 |
| Playing casino games at a casino |  |  |  |  |  |  | |  |  |  |  |  |  | 0.2 | 0.2 | -2.0 | 0.1 | 0.0 | 1.0 |
| Playing bingo at a club* |  |  |  |  |  |  | |  |  |  |  |  |  | 2.2 | 0.9 | 2.0 | 0.0 | 1.0 | 4.9 |
| Football pools |  |  |  |  |  |  | |  |  |  |  |  |  | 1.2 | 0.6 | 0.3 | 0.8 | 0.4 | 3.3 |
| Playing poker at a pub/club** |  |  |  |  |  |  | |  |  |  |  |  |  | 10.8 | 6.8 | 3.8 | 0.0 | 3.1 | 36.9 |
| Private betting or gambling with friends, family or colleagues |  |  |  |  |  |  | |  |  |  |  |  |  | 0.5 | 0.3 | -1.3 | 0.2 | 0.1 | 1.5 |
